# The Emotional Reinforcement Mechanism of and Phased Intervention Strategies for Social Media Addiction

**DOI:** 10.3390/bs15050665

**Published:** 2025-05-13

**Authors:** Jingsong Wang, Shen Wang

**Affiliations:** School of Physical Education and Sport Science, Fujian Normal University, Fuzhou 350108, China; qbx20230034@yjs.fjnu.edu.cn

**Keywords:** social media addiction, emotional reinforcement mechanism, positive and negative reinforcement, intervention strategy

## Abstract

Social media addiction has become a global public health challenge, and understanding its mechanism’s complexity requires the integration of the transitional characteristics of addiction development stages and breaking through the traditional single-reinforcement-path explanatory framework. This study is based on the dual pathway of positive and negative emotional reinforcement, integrating multidisciplinary evidence from neuroscience, psychology, and computational behavioral science to propose an independent and dynamic interaction mechanism of positive reinforcement (driven by social rewards) and negative reinforcement (driven by emotional avoidance) in social media addiction. Through a review, it was found that early addiction is mediated by the midbrain limbic dopamine system due to immediate pleasurable experiences (such as liking), while late addiction is maintained by negative emotional cycles due to the dysfunction of the prefrontal limbic circuit. The transition from early addiction to late addiction is characterized by independence and interactivity. Based on this, a phased intervention strategy is proposed, which uses reward competition strategies (such as cognitive behavioral therapy and alternative rewards) to weaken dopamine sensitization in the positive reinforcement stage, enhances self-control by blocking emotional escape (such as through mindfulness training and algorithm innovation) in the negative reinforcement stage, and uses cross-pathway joint intervention in the interaction stage. This study provides a theoretical integration framework for interdisciplinary research on social media addiction from a dynamic perspective for the first time. It is recommended that emotional reinforcement variables are included in addiction diagnosis, opening up new paths for precise intervention in different stages of social media addiction development.

## 1. Introduction

Social media encompasses content creation and sharing platforms based on user relations on the Internet, including but not limited to social networking sites, microblogs, WeChat, etc. Social media encompasses a diverse range of platform types, with significant differences in functionality and user interaction patterns. For example, with instant messaging and private social networking as its core, WeChat supports text, voice messages, and sharing on social media, with a focus on maintaining a social network of acquaintances; Instagram/TikTok focus on the creation and public dissemination of visual content (images, short videos), promoting user-generated content (UGC) and public participation and strengthening instant feedback through algorithm recommendations and ‘like’ mechanisms; and Weibo combines news dissemination with open social networking, with users being able to participate in public topic discussions through sharing and commenting. The designs of these platforms (such as the reward types and interaction frequency) may have different impacts on addiction mechanisms, and targeted exploration is needed in subsequent analysis. Social media provides many conveniences for people’s lives, such as helping with maintaining relationships with family and friends, finding companions, obtaining trending news, providing entertainment, sharing their lives, and expressing opinions through the use of social media platforms (Whiting & Williams, 2013). It is not difficult to see that, in addition to face-to-face interaction, social media has become the main channel for interpersonal communication in the digital age (Cheng et al., 2022). But with the gradual increase in the convenience, entertainment value, and popularity of social media use, the potential negative consequences it may bring have also gradually attracted public attention. Research has shown that the prevalence of social media addiction has reached 24% in 32 countries and regions (Cheng et al., 2021). Social media is as addictive as nicotine and has a profound impact on the mental health of adolescents during their growth and development stages (Mojtabai, 2024). It can easily lead to a series of negative consequences, such as anxiety (Dong et al., 2024), depression (Shanshal et al., 2024), emotional problems, and interpersonal alienation.

Scholars have conducted extensive research on social media addiction, aiming to explore its underlying mechanisms and seek effective intervention measures (Colasante et al., 2024; Julia et al., 2023; N. Zhao et al., 2024). However, research in this field is still in a relatively early stage, which inevitably increases the difficulty for people to have a clear understanding of social media addiction and greatly delays the process of clinical intervention. For this reason, since 2018, the European Internet Problem Use Network (European Research Group), which is composed of 146 members from 41 countries, has conducted intense discussions on the key issues facing problematic Internet use and issued a declaration (Fineberg et al., 2018). To summarize this, the first problem to be solved is to develop effective assessment tools (for different ages and socio-cultural backgrounds) or establish digital biomarkers to identify the severity and development process of addiction, so as to achieve early prevention and precise treatment. However, there is currently no officially recognized diagnostic standard for social media addiction, with the current options including the recently criticized Bergen Social Media Addiction Scale, which does not clearly distinguish between normal or prolonged use and pathological social media use (Fineberg et al., 2022). This can easily limit people’s focus to a certain stage of development of social media addiction (such as late-stage pathological manifestations), often ignoring the hidden characteristics of the dynamic transformation of emotional valence during the occurrence and development of addiction (i.e., the migration from being driven by positive emotions early on to late negative emotion avoidance). This weakens the consideration of an important characteristic of behavioral addiction that distinguishes it from substance addiction, which involves the damage caused by drugs to the brain structure and function, while behavioral addiction involves the continuous reinforcement of behavior driven by motivation (Zou et al., 2017). The empirical research on social media addiction is not yet mature, and most of the adjacent substance addictions and behavioral addictions are only studied at a certain stage of onset. Therefore, it is urgent to explore the characteristics of behavior reinforcement and symptom evolution at each stage of the development process of social media addiction, in order to incorporate behavior reinforcement variables into the diagnostic evaluation of addiction severity and development stages and increase the objective accuracy of social media addiction assessment, and based on this, targeted intervention plans will be developed for the different behavioral characteristics of each addiction stage, providing theoretical support for the prevention and clinical treatment of social media addiction.

As a typical hedonic information technology (Vaghefi et al., 2023), social media has a strong appeal similar to that of online games and can provide players with a sense of pleasure or psychological arousal (Granic et al., 2014). The pursuit of hedonic experiences is mainly achieved through two paths: obtaining satisfaction and reducing pain (Walia et al., 2022). The hedonic pursuit of this gaming experience can increase players’ subjective well-being in the short term, but long-term use can easily lead to the acquisition of addictive behaviors (Kringelbach & Berridge, 2009). The social feedback received through social media is both frequent and quantifiable (such as likes and followers), which causes both reinforcement and strong resistance to behavioral regression, making it highly susceptible to inducing addictive behavior (Greenfield, 2007). The component model proposed by Griffiths (Griffiths, 2005) suggests that emotional regulation is one of the key components of addiction, referring to individuals changing their emotional state by participating in addictive activities. Research has indeed found that game behavior driven by hedonism is often associated with negative consequences such as gaming disorders (Lee et al., 2021; Vaghefi et al., 2017). Solomon’s (1980) theory of adversarial processing suggests that when pleasurable experiences are triggered, the central nervous system maintains homeostasis through dynamic neural regulation mechanisms. The core of this theory includes two interactive processes: process a is responsible for processing the positive reinforcement from initial reward stimuli, and its function gradually weakens with repeated exposure; as a neural adaptive response, process b is gradually enhanced through a negative reinforcement mechanism. This neural adaptation mechanism is manifested as a decrease in the activity of process a and a gradual dominance of process b with continuous exposure to the same stimuli, ultimately leading to the development of addictive behavior (Koob, 2009). From this, it can be seen that emotional changes may be a key mechanism for the occurrence and development of addiction. Therefore, exploring the internal mechanisms of social media addiction based on emotional regulation, especially the path of enhancing positive emotions and reducing negative emotions, may be an important way to deeply reveal the processes behind the occurrence and development of social media addiction. At the same time, it is helpful for people to explain the complexity of and individual differences in social media addiction.

The existing theoretical models on Internet use issues (I-PACE model, component model, etc.) have made great progress, but for social media addiction, one of their key subtypes, we still lack an in-depth analysis of dynamic neurobehavioral mechanisms. At the same time, in the context of the Internet in this new era, recent technologies are emerging endlessly (such as big data algorithms, Akhund et al., 2024), and some of the theoretical models proposed in the past 20 years seem somewhat outdated (Moretta et al., 2022). Therefore, to accurately explain the problem of social media addiction, this study integrates the existing empirical research and theoretical basis with the background and technology of the Internet in this new era and deeply discusses how a positive and negative emotional reinforcement mechanism can promote the occurrence and development of social media addiction, so as to build a corresponding theoretical model and make up for the lack of an explanation given by traditional models regarding the dynamic changes in emotional potency. Finally, based on this, corresponding intervention strategies are proposed in order to provide a theoretical reference for frontline clinical workers.

## 2. Conceptualization of Social Media Addiction

Social media addiction is a social psychological phenomenon characterized by the prolonged and high-intensity use of social media, leading to an irresistible extension of usage time and resulting in psychological distress and physiological discomfort (Moreau et al., 2015). The manifestations of social media addiction vary depending on the platform’s functionality. For example, an addiction to WeChat may manifest as the frequent checking of messages or updates in social circles to maintain social relationships (privacy enhancement), while addiction to Instagram/TikTok is more related to visual content creation, like-chasing, and algorithm-driven infinite browsing (openness enhancement). This difference requires the construction of theoretical models to consider the applications of the platform. Some researchers believe that (Douglas et al., 2008; Milani et al., 2009) the overuse of social media is closely related to Internet-based addiction behavior and the overuse of social media can produce similar negative psychological and physiological consequences to those of game addiction and Internet addiction (Gosling et al., 2011; Ross et al., 2009). There is research supporting this viewpoint (Kuss & Griffiths, 2011), and it is believed that the excessive use of social media is another form of online activity that leads to addictive behavior in adolescents, such as that described by the diagnostic criteria for Internet addiction based on the American Psychiatric Association’s DSM-V (American Psychiatric Association, 2013); this phenomenon can be called social media addiction. But some researchers believe (Laconi et al., 2014) that the overuse of social media simply results in psychological and behavioral discomfort caused by the improper or prolonged use of social media and that its symptoms have not yet reached the level of addiction, which is equivalent to the early stages of addiction development, where an individual is not yet in a pathological condition. It has significant differences from a psychiatric addiction disorder, with a psychiatric addiction disorder mainly being characterized by pathological psychological and behavioral symptoms that deviate from the norm, while the overuse of social media is mainly characterized by normal psychological and behavioral symptoms that deviate slightly from the norm. Meanwhile, social media addiction belongs to the category of behavioral addiction, which is both distinct and related to gambling addiction and substance addiction (Zou et al., 2017). Both behavioral addiction and substance addiction involve the dysfunction of the midbrain limbic dopamine system, particularly in the reward circuits of the nucleus accumbens and anterior cingulate cortex (Kuss & Griffiths, 2012). Social media addiction is also similar to gambling addiction, with both manifesting as increased tolerance (requiring increasing usage time), withdrawal symptoms (such as anxiety and irritability), a loss of control (inability to reduce usage), and functional impairment (Griffiths, 2005). But the difference is that social media generates intermittent reinforcement mechanisms through instant interactive feedback (such as likes and comments), and this social reward may be more sustainable than the monetary reward of gambling (W. Zhou et al., 2019). In addition, cultural factors have a more significant impact on social media addiction, such as social pressure in collectivist cultures, which may exacerbate usage behavior (Zou et al., 2017).

The above controversy actually reflects the phased characteristics of addiction development. Theoretical models in the field of behavioral addiction, such as the cognitive behavioral model proposed by Davis (2001), can to some extent be used to understand the occurrence and maintenance process of gaming disorders. At the same time, Davis believes that pathological Internet use is caused by “problematic cognition plus reinforcement and maintenance of bad behavior”. The I-PACE model proposed by M. Brand et al. (2019) also divides the addiction process into early and late stages, which have different cognitive, emotional, and executive function characteristics. This study attempts to construct an operational definition framework which preliminarily defines social media overuse behavior (commonly referred to as “problematic use” in the literature) that has not yet fully met pathological diagnostic criteria but has caused functional impairment as the early stage of addiction, while pathological social media use that meets strict clinical criteria is classified as the late stage of addiction. The early behavioral manifestations of social media addiction should be based on normal social media use, with individuals being in a state between pathological and normal use. This state carries the risk of transitioning from normal use to pathological use and can further predict late-stage behavior in social media addiction. The term ‘Normal Use’ referred to here means, according to Luo et al., that an individual’s Social Media Addiction Scale (BSMAS) score is below 24 points (Luo et al., 2021), while J. Yao (2017) defined the behavior and concept of normal use as a moderate decrease in users’ level of social network use, manifested as users actively reducing the time and frequency of their social network use. This is also similar to Brand’s description of the use of functional networks as a tool to handle daily needs and goals in a healthy way (M. Brand et al., 2014). It should be noted that there is still no clear diagnostic boundary for social media addiction, and this classification is not an absolute ternary division (normal, risk, and addiction). Therefore, the validity of the stage division needs to be further validated through future longitudinal studies.

## 3. The Potential Role of Emotional Regulation in the Development of Social Media Addiction: The Driving Role of Positive and Negative Reinforcement 

Emotion regulation refers to the process in which an individual attempts to change the intensity, duration, frequency, and category of their current or expected emotional state (Gross, 2002). Emotional regulation requires two processing abilities: an individual’s emotional regulation ability and the ability to use emotional regulation strategies. Research has shown that both an impaired emotional regulation ability and the impaired use of emotional regulation strategies are significantly associated with numerous mental and psychological disorders, including anxiety, depression, substance abuse, eating disorders, gambling disorders, and gaming disorders (Aldao et al., 2010; Estévez et al., 2017; Sheppes et al., 2015); this indicates that emotional disorders may be a cross-diagnostic disease risk factor. From a functionalist perspective, research has also found that the development and maintenance of numerous maladaptive behaviors, such as self-harm, overeating, chronic anxiety, substance abuse, and clinically impulsive and compulsive behaviors, are driven by emotional regulation itself (Swerdlow et al., 2020). Emotions are closely related to motivation. Motivation can generate or exacerbate emotional arousal, while emotions can create, strengthen, or weaken motivation. Happy experiences can motivate people to participate in various behaviors they have learned or expect to be enjoyable (Cameron & Bernstein, 2022). Therefore, exploring the emotions associated with social media addiction can help deepen our understanding of the mechanisms underlying the occurrence and maintenance of social media addiction and provide a theoretical and empirical basis for possible intervention measures in the future. The existing research on social media addiction mainly focuses on cross-sectional and longitudinal studies on emotions (Colasante et al., 2024; Xiao et al., 2022; Rogier et al., 2024). There are few empirical studies on the emotional mechanisms of social media addiction, which further limits the in-depth exploration of its underlying mechanisms. Therefore, there is an urgent need for theoretical models to provide guidance and reference on how emotional regulation impairment can promote the occurrence and maintenance of social media addiction. Research and theories related to addiction fields such as substance addiction or behavioral addiction can provide us with references for summarizing and exploring the mechanisms of such theories, as previous studies have found that substance addiction and behavioral addiction, including gambling and gaming disorders, have similar underlying mechanisms (M. Brand et al., 2014; Weinstein & Lejoyeux, 2015; Yau & Potenza, 2015).

The explanation of positive and negative reinforcement is considered the most intuitive explanation for addiction and has long dominated addiction-related theories (Robinson & Berridge, 2003; Wise & Koob, 2014). According to Skinner’s operant conditioning reflex principle, the R-O linkage formed between the behavior of an organism and its subsequent behavioral consequences plays an important role in the acquisition of instrumental behavior. R-O associative learning embodies the principle of positive and negative reinforcement. Specifically, the pleasurable outcomes brought about by an organism’s behavioral response can increase the frequency of that behavioral response, demonstrating the role of positive reinforcement. The disappearance of aversive stimuli after an organism’s behavioral response increases the frequency of behavioral responses, reflecting the effect of negative reinforcement. In relation to the occurrence and development process of addiction, the positive reinforcement mechanism involves the enhancement of positive emotions through participation in addictive behaviors, while the negative reinforcement mechanism involves the alleviation or elimination of negative emotions through participation in addictive behaviors (Brewer & Potenza, 2008; Lee et al., 2014), essentially reflecting the changes in individual emotional states during the addiction process. Therefore, researchers often explore the process of addiction from the perspective of hedonism and propose the “hedonic management model of addiction”, which includes the consideration of positive and negative reinforcement. It is also believed that the basic process of addictive behavior involves the satisfaction of a psychological state regulation function, the aim of which is to obtain a certain sense of satisfaction or avoid pain (Brown, 1997). Therefore, in a sense, addictive behavior itself may reflect a non-adaptive emotion regulation strategy, which is consolidated through positive and negative reinforcement mechanisms, forming the basis of the addiction process (M. Brand et al., 2016, 2019). Behavioral manifestations include the excessive use or participation in certain substances or activities, thereby achieving the goal of changing or regulating an individual’s internal emotional state. Previous studies on substance addiction and behavioral addiction have only explored the development and maintenance of social media addiction through a single reinforcement pathway (such as simple positive or negative reinforcement). If we explore the development and maintenance of social media addiction from the perspective of dual pathways of emotional positive and negative reinforcement, it can help people better understand the complexity of social media addiction diseases and consider individual differences, prevent problems before they occur, and treat them accurately.

## 4. Dual-Pathway Mechanism of Positive and Negative Emotional Reinforcement in Social Media Addiction: Empirical Evidence and Theoretical Basis

### 4.1. Positive Reinforcement Pathway

Social media allows for immediate access to social information when needed (Nesi et al., 2018; Odgers & Jensen, 2020; Prinstein et al., 2020) and maintains user engagement by maximizing social rewards. Likes, notifications, and messages arrive unpredictably with the most powerful variable reinforcement schedule, making individuals habitually check social media in anticipation of this social feedback (Griffiths, 2018). Repeated exposure to digital social rewards (such as notifications or likes) may increase neural responses to reward-related cues, thereby reducing adolescents’ ability to resist the impulse to view social media (Veissière & Stendel, 2018; Ihssen & Wadsley, 2021). Neuroimaging studies have shown that social media interaction, especially liking behavior, can significantly activate the striatum (the core brain region of the dopamine system), and the intensity of activation in this area is dose-dependently positively correlated with subjective pleasure (Sherman et al., 2018). This mechanism is further confirmed by behavioral evidence, specifically that social media platforms significantly increase their use frequency and behavioral stickiness through “variable ratio reinforcement” (intermittent and unpredictable reward designs similar to those of gambling) (Clark & Zack, 2023). From the perspective of neurotransmitters, the dopamine system plays a dual regulatory role in social reward sensitivity: on the one hand, dopamine D2 receptors amplify the value evaluation of social feedback by regulating the motivational process and motivational significance of social behavior (H. Chen et al., 2024; Robbins & Everitt, 2007; Salamone & Correa, 2012), while on the other hand, the administration of dopamine antagonists can significantly inhibit individuals’ behavioral tendency to pursue social rewards (Salamone & Correa, 2012). These findings collectively suggest that social rewards induce immediate pleasurable experiences by activating the midbrain limbic dopamine pathway, which forms the neurobehavioral basis of the positive reinforcement pathway (Haber & Knutson, 2010). Therefore, some studies have suggested that positive emotional changes (positive reinforcement) caused by changes in emotional regulation may mainly affect the early stages of addiction, and long-term dependence may shift towards maintaining negative emotions (negative reinforcement) (Kuss & Griffiths, 2017). This dynamic evolution suggests that interventions for social media addiction need to differentiate between reinforcement mechanism targets at different stages. It is worth noting that the positive emotional experience during the positive reinforcement stage may be due to the unique attributes of online social interaction (immediacy, convenience, anonymity) that expand the reward sensitivity window and accelerate the addiction process.

The theoretical explanation of this pathway involves a multidisciplinary framework; specifically, according to the I-PACE model (M. Brand et al., 2019), the interaction between emotions and cognition generates positive reinforcement expectations (such as positive expectations of social rewards), which can significantly enhance the tendency towards repeated use behavior. This model suggests that positive emotional experiences (such as happiness and satisfaction) and cognitive biases (such as “like prediction”) jointly reinforce the “use reward” cycle, forming the psychological motivational basis for addictive behavior (Julia et al., 2023). The Social Reward Sensitivity Theory (Haber & Knutson, 2010) reveals that the striatum drives behavioral motivation by encoding social recognition value (such as that provided by likes and follows), and its neural activity intensity can predict an individual’s degree of social media dependence. The motivation sensitization theory (Robinson & Berridge, 1993) provides a dynamic explanation of how long-term social media exposure can lead to dopamine system sensitization, causing addicts to have an excessive attentional bias towards social cues (such as message cues) and behavioral convergence. The design logic of social media is deeply rooted in the theory of intermittent reinforcement (H. T. Zhang, 2023), where platforms continuously stimulate dopamine release through unpredictable reward placements (such as randomly appearing likes and the pushing of popular content), exacerbating reward prediction errors and gradually transforming social interaction from functional behavior to addictive behavior (Meshi et al., 2015).

### 4.2. Negative Reinforcement Pathway

The essence of the negative reinforcement pathway lies in individuals using social media to escape or alleviate immediate negative emotions such as loneliness, stress, and social anxiety (Wolfers & Utz, 2022; Turhan Gürbüz et al., 2021; G. Shen et al., 2024), which may enhance addictive behavior due to pain relief effects. A recent review on addictive behaviors such as gaming (Melodia et al., 2020) identified avoidance motivation (i.e., evading reality, especially real-world problems such as daily troubles and unpleasant situations (Demetrovics et al., 2011)) and coping motivation (i.e., dealing with (dispersing) stress and improving negative emotions) (Demetrovics et al., 2011) as the most common motivational factors for technology addictions, such as IGD. In addition, previous studies have shown that avoidance is one of the main motivations for a person to use TikTok (Gu et al., 2022). Longitudinal studies have shown that regardless of baseline stress levels, the intensity of the negative reinforcement process can significantly predict the deterioration trajectory of addictive behavior (Moretta et al., 2023). In addition, a cross-sectional study showed that undergraduates often used social media to make online social contact with others to regulate negative emotions during isolation due to COVID-19 (Michikyan et al., 2023). At the same time, neuroimaging studies have provided a biological mechanism explanation for this; that is, compared with low-frequency users, high-frequency users of social media exhibit a decline in their prefrontal cortex (PFC) control function, specifically manifesting as a decrease in their gray matter volume and the weakened activation of inhibitory control-related brain areas (such as the dlPFC) (Achterberg et al., 2022). This neuroadaptive change may make it difficult for individuals to suppress compulsive use impulses, forming a vicious cycle of “emotional avoidance self-regulation failure”. It is worth noting that a fear of missing out (FoMO), as a psychological driving force of negative reinforcement, is dose-dependently associated with the degree of social media addiction (Montag et al., 2023). Clinical studies have further revealed that individuals with a tendency towards depression can temporarily alleviate symptoms using social media for short-term emotional regulation (such as shifting attention), but social comparison and self-evaluation threats exacerbate emotional deterioration (Lin et al., 2016). Meanwhile, if an individual’s expectations for social media use are not met, in order to make up for the unfulfilled expectations, users may adopt some high-risk behaviors, such as posting more controversial content or excessively pursuing attention (Turel & Qahri-Saremi, 2024), which will further trigger negative reinforcement effects. Although negative emotions (anxiety, depression, etc.) are important factors in the development of social media addiction, the relationship between the two is not a single pathway through which negative emotions affect social media addiction. Especially in the late stages of social media addiction development, withdrawal symptoms such as irritability and anxiety (Kaptsis et al., 2016), a fear of missing out (the content presented on social media is often exaggerated or glorified, exacerbating people’s desire for good experiences, leading to social comparison, jealousy, a feeling of loss, and other psychological states that can exacerbate a fear of missing out) (N. Wang et al., 2023), self-control failure (Garcia & Cooper, 2024), etc., also contribute to the evolution of negative reinforcement mechanisms to some extent. Thinking dialectally about the relationship between negative emotions and social media addiction is beneficial for developing more reasonable intervention strategies. Physiological experiments have supported this bidirectional effect, where individuals with high addiction tendencies experienced significantly higher levels of cortisol after exposure to social stressors compared to the control group. This suggests that the overactivation of the hypothalamic–pituitary–adrenal axis (HPA axis) may mediate compulsive use behavior (Shafi et al., 2021), which also reflects the biological association between stress and compulsive use. Although social media itself can be used as a regulatory tool for stress responses, it is worth noting that while using Facebook can temporarily inhibit cortisol secretion, the cost may be the increased risk of relapse (Rus & Tiemensma, 2018).

According to the I-PACE model (M. Brand et al., 2016), individuals’ negative reinforcement expectations of social media use (i.e., the expectation that it can reduce negative emotions) drive addictive behavior through the interaction of emotion regulation and cognitive bias. The model suggests that when users view social media as the default strategy for coping with stress, their usage behavior is reinforced by short-term emotional relief, while weakening their adaptive coping abilities in reality (such as problem-solving and seeking social support). The Social Skill Deficiency Vulnerability Hypothesis (a theory used to conceptualize the maintenance of social anxiety) (Scott, 2005; Davis, 2001) suggests that individuals with insufficient social skills, such as those with social anxiety or low self-disclosure efficacy, are more likely to rely on online interaction instead of actual social interaction (Corina et al., 2024). Although this compensatory use pattern can reduce social threat perception due to anonymity and controllability, it may further lead to the degradation of social skills and form a vicious cycle over time. In addition, theoretical support for the negative reinforcement pathway comes from the avoidance model (Baker et al., 2004) and the addictive negative reinforcement neural framework (Koob & Volkow, 2016), where the former emphasizes that behavior is reinforced by reducing negative emotions, while the latter suggests that long-term stress leads to changes in neural plasticity and a long-term dependence on negative reinforcement may lead to the degeneration of prefrontal function, forming a vicious cycle of “use function impairment stronger dependence”. Therefore, based on the above models and empirical research definitions, when individuals solidify their social media use as an exclusive mechanism for relieving stress, loneliness, or depression, their behavioral patterns are prone to deviating from the normal track of self-control due to negative reinforcement effects (short-term emotional relief) and neural adaptation (long-term decline in control function). This process is accompanied by the exhaustion of psychological resilience and the weakness of alternative coping strategies, ultimately leading to a pathological dependence on social media use.

### 4.3. Interaction Between Positive and Negative Reinforcement Pathways

The dual positive and negative reinforcement pathways operate independently and interact dynamically (L. Yao et al., 2017). From the perspective of microscopic synaptic plasticity (Kravitz & Kreitzer, 2012), long-term potentiation (LTP) and long-term depression (LTD) constitute the molecular basis of positive and negative reinforcement learning, respectively. Positive reinforcement induces LTP through the activation of the striatal direct pathway, promoting reward-seeking behavior (such as continuous refreshing dynamics), while negative reinforcement triggers LTD through indirect pathways, enhancing avoidance behavior (such as passive browsing to avoid reality) (Kravitz & Kreitzer, 2012). This pathway-specific synaptic plasticity differentiation lays the molecular biology foundation for the functional independence of the two systems. In terms of neural circuits, X. Liu et al.’s (2011) meta-analysis of 142 brain imaging studies revealed that positive reinforcement (such as receiving likes) mainly activates the core brain areas of the reward network (nucleus accumbens, medial orbitofrontal cortex, posterior cingulate cortex), which may mediate positive emotional experiences (happiness, satisfaction). Negative reinforcement (such as alleviating loneliness) simultaneously activates the reward network (nucleus accumbens) and stress-monitoring network (anterior cingulate cortex, anterior insula, dorsolateral prefrontal cortex), which can easily trigger negative emotion regulation. It can be seen that the brain regions involved in positive and negative reinforcement learning are both overlapping and distinct, such as the striatum and thalamus (X. Liu et al., 2011). Therefore, some scholars have further conducted research on the neural mechanisms of positive and negative reinforcement, but the results have shown that although positive reinforcement and negative reinforcement have different activation and inhibition pathways, there may be an interaction between the two pathways (L. Yao et al., 2017). For example, studies have shown that negative reinforcement plays a role in the early stages of addiction (May et al., 2020), while the dopamine system responsible for reward sensitivity also plays an important role in the late stages of addiction (Yang, 2023). It can be seen that although the positive and negative reinforcement pathways are independent of each other, the positive reinforcement stage does not only involve positive emotional reinforcement, and the negative reinforcement stage does not only involve negative emotional reinforcement. At the same time, the positive and negative reinforcement pathways of social media addiction are easily and rapidly transformed under the influence of certain factors, resulting in both positive reinforcement effects (highly unstable) and negative reinforcement effects of social media at a certain stage. For example, when users receive social rewards (such as likes and comments) that are likely to trigger positive reinforcement effects, if their value is lower than their self-expectations (when they do not receive likes or comments from good friends), they may feel neglected or rejected, causing emotional pressure and triggering negative reinforcement effects (Angelini & Gini, 2025). But there is also another scenario where users can automatically detach themselves from certain social media behaviors after receiving lower-than-expected social rewards, which may be due to the computational framework of reinforcement learning (RL), which consists of three stages: updating the expected rewards, evaluating the expected returns by considering the subjective costs (such as effort), and choosing actions (Turner et al., 2024). In order to motivate daily social behavior, we assign value to achieving immediate goals, but when this value cannot meet individual needs, people evaluate the cost and effort to choose alternative actions. This is not the same as the repetitive behavior of positive reinforcement. In addition, the viewpoint that positive and negative reinforcement exhibit a phased transition has also been validated by scholars, and this dynamic characteristic plays an important role in the development of addiction, laying the foundation for people to better understand the evolution of addiction stages. For example, Koob and Volkow (2010) explored the roles of reward systems (such as the dopamine system) and negative emotion systems (such as stress responses) in the evolution of addictive behavior. Their study systematically elucidated the transition of addictive behavior from being reward-driven (positive reinforcement) to negative emotion-driven (negative reinforcement). After the positive pleasure from positive reinforcement occurs, the dominant negative avoidance due to negative reinforcement will appear immediately. However, the onset and progression of negative reinforcement process are slow, and the severity of the addiction will continue to worsen with repeated exposure to cues. Therefore, the above research systematically elucidated the paradigm of the independence, interaction, and dynamic transition between positive and negative reinforcement pathways in social media addiction. In summary, the positive and negative reinforcement pathways are both independent of each other and have interactive effects. The characteristic of a phased transition is present through the entire addiction process, which also shapes the complexity of social media addiction.

**Figure 1:**
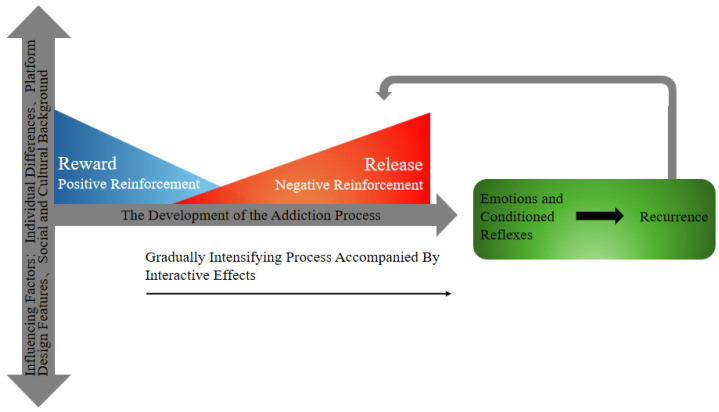
Conceptual framework diagram of positive and negative reinforcement-driven development of social media addiction.

The theoretical basis can also draw on models related to substance addiction and behavioral addiction. For example, the three-stage model of drug addiction suggests that addiction represents a severe disruption of the motivational circuit, characterized by three stages: (1) binge eating/poisoning, driven by reward motivation, involving changes in dopamine and opioid peptides in the basal ganglia; (2) withdrawal/negative effects, driven by fear motivation to avoid negative emotions, involving a decrease in dopamine function and an increase in stress neurotransmitters in the brain, leading to stress in the amygdala circuit; (3) and prejudices and expectations characterized by habitual motivation to consume addictive substances, involving the dysregulation of the prefrontal cortex and insula, affecting the basal ganglia and amygdala, and leading to craving and deficits in executive function (Koob & Volkow, 2016). This shift from pursuing rewards and avoiding discomfort to habit-driven compulsive behavior lays the foundation for understanding the process of social media addiction (Koob & Volkow, 2016). The I-PACE model in the field of behavioral addiction (M. Brand et al., 2019) distinguishes between the early and late stages of the addiction process. Addictive behavior exhibits a phased evolution, initially dominated by positive reinforcement (seeking likes, social recognition) and often accompanied by impulsive behavior. As tolerance develops, it gradually shifts towards the dominance of negative reinforcement (avoidance of loneliness, withdrawal anxiety) and is accompanied by compulsive behavior. This indicates that positive and negative reinforcement are independent of each other but have a transitional characteristic. The theory of oppositional processes explains how addictive behavior shifts from pursuing rewards to avoiding negative emotions (Koob & Le Moal, 2008). The social homeostasis hypothesis suggests that the brain can maintain adaptive behavior by dynamically balancing social rewards with potential threats (such as exclusion risks), thereby promoting the development of social behavior (Isaac & Murugan, 2024). The dynamic system theory emphasizes the dynamic interaction between individual behavior and the environment. In social media addiction, the positive and negative reinforcement pathways constitute a dynamic equilibrium system, which is regulated by individual traits (such as impulsivity), environmental stress (such as social exclusion), and platform algorithms (such as those creating an affective push) in a triple manner (Yan, 2025). This dynamic equilibrium system constantly adjusts according to the interaction between individuals and the environment, ultimately leading to the formation and maintenance of addictive behavior. Of course, the currently described interaction between the positive and negative reinforcement pathways of social media addiction has only been theoretically derived and constructed. In the future, longitudinal empirical research is needed to provide direct evidence support. The current theoretical model is only for scholars to refer to and use as a reference. Please be cautious and provide empirical evidence. A conceptual framework diagram of the positive and negative reinforcement-driven development of social media addiction is shown in Figure 1.

## 5. Influencing Factors: Variables That Regulate the Strength of the Dual Positive and Negative Reinforcement Pathways

### 5.1. Individual Differences

Personality traits and pathological psychological states are potential risk factors for social media addiction (Treviño Benavides et al., 2023). Individuals with high extroversion and peer popularity are more sensitive to positive reinforcement, and their social reward needs drive frequent use, while individuals with high neuroticism and impulsivity rely more on negative reinforcement and escape the pressure of reality through social media (N. Zhao et al., 2024; W. Wang, 2018). Psychopathological factors further exacerbate pathway imbalances, with individuals with depressive tendencies experiencing an increased frequency of negative reinforcement use (Feng et al., 2025), while individuals with social anxiety are trapped in a cycle alternating between the dual pathways due to their “online safety dependence” (Du et al., 2024). Cognitive dysfunction, such as inadequate self-control, can also weaken the ability to regulate the dual pathways and accelerate the transition from positive reinforcement to negative reinforcement. This phenomenon is particularly evident in individuals who excessively use social media, while negative emotions increase social media use by reducing self-control (L. Zhang, 2024).

### 5.2. Platform Design Features

Needs and motivation are key personal factors that affect SMA (Liang et al., 2024). For example, individuals with a self-presentation motivation are more eager to showcase a more prosperous and popular self (Andreassen et al., 2017; A. Chen, 2019; C. Liu & Ma, 2019). The interactive features of social media perfectly meet users’ self-presentation needs (Liang et al., 2024). Some positive reinforcement designs, such as variable reward mechanisms (infinite scrolling and personalized recommendations), can activate the dopamine system (Clark & Zack, 2023). At the same time, the innovation of social monetization, such as quantifying the number of fans and amount of interaction using visual indicators (fan progress bars), can greatly enhance reward perception (C. Zhang et al., 2023). Some negative reinforcement designs, such as the FOMO algorithm, enhance users’ anxiety by pushing notifications such as “friends are viewing”, triggering avoidance behaviors (such as frequently refreshing or checking social media) to alleviate this anxiety (Y. F. Zhang et al., 2020). At the same time, sentiment computing can also prioritize the recommendation of entertainment content to promote evasion when detecting users’ low mood (through speech or text analysis) (Akhund et al., 2024). This emotional push design can effectively prolong the dual-pathway cycle.

### 5.3. Social and Cultural Background

Different socio-cultural backgrounds are an important factor influencing social media addiction (Ong & Lee, 2022). Individuals from East Asian cultures tend to showcase their achievements and display a positive image using social media in order to gain recognition and respect from others (Zheng & Tan, 2022). Meanwhile, social media addiction exacerbates negative reinforcement mechanisms through social comparison pressure, particularly in collectivist cultural contexts where this pressure is more pronounced, leading to higher levels of depression (Y. Zhao & Huang, 2023). In individualistic cultures, there is a growing emphasis on showcasing individual achievements. For example, professional social platforms such as LinkedIn strengthen individuals’ pursuit of career success through features such as skill tags and career achievement displays. Individuals in this cultural context tend to gain social recognition and psychological satisfaction by showcasing their achievements (Y. Shen, 2024). Negative reinforcement in individualistic cultures is more significantly associated with feelings of loneliness. Research has shown (McPherson et al., 2006) that although social media provides a wide range of social interaction opportunities, this interaction often lacks depth, leading individuals to feel lonelier. For example, research in the United States has found that the higher the frequency of social media use, the stronger the sense of loneliness individuals feel (McPherson et al., 2006). Religious and ethical norms are also important considerations, such as in conservative cultures (such as the Middle East), where social media anonymity amplifies negative reinforcement use (Li, 2023) (evading the social constraints of reality).

## 6. Intervention Strategies: Targeted Programs Based on Positive and Negative Reinforcement Pathway Characteristics

### 6.1. Intervention Strategies Based on Positive Reinforcement Pathway Features

Targeted interventions targeting the excessive sensitization of the dopamine reward system during the positive reinforcement stage may provide some therapeutic benefits for people. For example, regarding cognitive behavioral therapy (Young, 2007; Gupta et al., 2013), cognitive restructuring and behavioral experiments were conducted to correct the reward expectation biases of social media addicts (such as “likes determine self-worth”), guide users to reduce their posting frequency and observe emotional changes, and reduce their dependence on social rewards. At the same time, offline social reinforcement can be achieved through alternative reward training, such as by providing alternative social rewards through group activities (such as sports clubs) (Jones, 2024). Exercise, as a natural reward, can alter the midbrain limbic dopamine pathway and prefrontal emotion assessment circuit (Greenwood, 2019), thereby increasing natural rewards and antagonizing social media addiction rewards. Group activities involving simultaneous exercise can generate experiences of emotional peaks and steady emotional states (Qu et al., 2017; H. Zhou & Wang, 2009), which can to some extent achieve a neural representation substituting for pleasure from virtual rewards. However, it is recommended that people in the positive reinforcement stage of social media addiction engage in fun and low-intensity aerobic exercise, as studies have shown a correlation between low physical activity and long periods of sitting and the degree of mobile phone addiction (Ren et al., 2022). For those who have no exercise habits or experience, the lactic acid accumulation and muscle pain experienced due to directly engaging in high-intensity exercise often have the opposite effect to alternative reward training, which is not conducive to the formation of positive emotions and the cultivation of exercise interest (reduced compliance) (Maltagliati et al., 2024). In addition, through digital disconnection challenges or setting social media-free periods, individuals can replace virtual rewards with real achievements (such as learning new skills) to help them achieve more lasting satisfaction and intrinsic motivation. Research has shown that real-life achievements can not only provide long-term satisfaction but also replace the short-term pleasure brought by virtual rewards (X. L. Zhou et al., 2017).

### 6.2. Intervention Strategies Based on Negative Reinforcement Pathway Characteristics

Designing intervention strategies based on the characteristics of the negative reinforcement stage should not only ensure their effectiveness in alleviating emotional avoidance and stress reactions but also ensure that the intervention strategy can enhance the self-control ability of the prefrontal cortex. Behavioral training such as mindfulness meditation can increase the function of the control network, improve the function of the emotion regulation network, and reduce the avoidance of negative emotions, making it an effective method for treating addiction (Tang et al., 2016). Metacognitive intervention can help users effectively regulate and cope with stress and reduce avoidance behavior (Zhi, 2023). Cognitive behavioral therapy can enable participants to replace negative behavior with structured goals and positive thinking and provide motivation (Chand et al., 2023). At the same time, moderate-intensity aerobic exercise can effectively enhance the inhibitory control ability of individuals with an Internet addiction (Fan et al., 2021), promote rapid recovery from negative emotions caused by stress, improve emotional flexibility (Bernstein & McNally, 2017), and effectively alleviate negative moods and reduce their rumination level (S. Brand et al., 2018), but it is recommended to engage in moderate-intensity or above aerobic exercise, as otherwise it is difficult to cause changes in the brain inhibitory control function (Fan et al., 2021). Finally, social media platforms should be encouraged to strengthen responsible innovation, collaborate with academic researchers and community partners, customize algorithms, reduce the deployment of emotional algorithms, and create a health-focused environment for people (Russell et al., 2023).

### 6.3. Integrated Intervention Strategy Based on Interaction Characteristics of Positive and Negative Reinforcement Pathways

Personalized solutions should be developed based on the non-linear interaction characteristics of the dual positive and negative reinforcement pathways, achieving dynamic evaluation and hierarchical intervention. Treatments for early addiction should focus on positive reinforcement interventions (such as reward substitution), while treatment for long-term dependence should shift towards negative reinforcement management (such as mindfulness training). If there is a phenomenon of positive and negative reinforcement interaction, the clinical evaluation of social media addiction should be scientifically optimized prior to designing interventions. Although there is currently no officially recognized diagnostic evaluation standard for social media addiction, the proportion of positive and negative reinforcement in each stage of addiction development should be quantified as much as possible before designing personalized intervention plans. Firstly, it is recommended to identify dominant reinforcement mechanisms through latent category analysis (subjective scale evaluation) based on clinical interviews. Then, based on this, a combination therapy can be used for intervention. Research has shown that combined interventions are more effective than a single type of intervention, and exercise is considered the best single and auxiliary intervention method (Z. Zhou et al., 2024), but it is recommended that people with positive and negative reinforcement interactions increase their exercise intensity gradually from low to high. Finally, it is recommended to strengthen privacy protection for cross-platform data collaboration, avoid the leakage of users’ private data (C. Zhao et al., 2025), provide users with a safe and secure network usage environment, and create a minimally triggering environment for clinical intervention.

## 7. Future Research Directions

Social media addiction, as an important subtype of Internet addiction, is widely known but still in the early stages of research, with limited empirical studies and unclear pathways of action, which adds great difficulty to clinical treatment. Therefore, future research needs to deepen the understanding of the dynamic interaction mechanism and translational application of the dual pathway model, with a focus on the following: (1) Incorporating emotional positive and negative reinforcement variables (situational and emotional states associated with social media use) into the assessment of clinical severity and stage diagnosis based on this theoretical model: for example, clinical interviews combined with subjective addiction scales and the assessment of situational states (using fragmented time to quickly share updates during daytime work or study breaks to obtain feedback/frequently browsing social media platforms in the late evening to seek comfort) and emotional states (wanting to engage in interactions when happy and excited/wanting to escape reality when anxious and frustrated) associated with social media use can be used for potential profile analysis to increase the supporting evidence. (2) Neural circuit analysis can be incorporated and combined with multimodal brain imaging (such as fMRI and EEG) to elucidate the spatiotemporal encoding rules of the prefrontal striatum amygdala circuit in dual-pathway switching, especially regarding the functional mechanism for competition between the default mode network (DMN) and reward network. (3) Real-time monitoring technology development, utilizing artificial intelligence-driven digital phenotype analysis (such as speech emotion recognition and eye tracking) and ecological instant assessment (EMA), could allow us to capture the micro dynamics of positive and negative reinforcement motivations and their environmental triggering factors (such as stress events and algorithm pushes). (4) Cross-cultural adaptability verification could be conducted, establishing a dual-pathway weight model considering the influence of non-Western cultural backgrounds, such as the unique driving force of social recognition anxiety in negative reinforcement in East Asian “face culture” or the moderating effect of religious norms in the Middle East on dual-pathway expression patterns. (5) Precision intervention innovation could explore personalized therapies based on neural regulation (such as transcranial magnetic stimulation targeting the dlPFC) and gene typing, and the design of “ethical algorithms” could reduce the exploitative use of dual pathways in platform design (such as by dynamically adjusting the variable reward frequency). (6) Longitudinal tracking and prevention strategies could be employed to identify early biological behavioral markers of adolescent dual-pathway sensitivity through birth cohort studies (such as infant dopamine receptor gene expression and childhood social-feedback-seeking behavior) and to construct a developmental cascade model to guide interventions during the golden window period. In addition, teenagers are in a sensitive period of development, and their emotional issues should be given more attention. How the imbalance in the development of their impulse and control systems leads to the positive and negative reinforcement of social media addiction will be the focus of future research. These directions will drive the evolution of the dual-pathway theory from a descriptive framework to a computable and interventionable translational science, providing solutions to the public health challenges of global digital life.

## 8. Research Shortcomings and Limitations

This study mainly discussed the early and late development stages of social media addiction, based on the I-PACE model. Although this theoretical model mainly comprises positive and negative reinforcement pathways, the four factors of individual differences, emotions, cognition, and executive function are all reflected in this theoretical model. However, other frameworks with a similar focus on emotional motivation, such as those focused on goal orientation and habit systems, were less explored in this study. In the future, more theoretical frameworks will be included in detailed discussions and empirical research to increase the supporting evidence for this theoretical model. In addition, the literature reviewed had shortcomings and differences in terms of the definitions and structures examined, sample characteristics (sample convenience), and focus on Internet addiction in the research. These studies covered a wide range of behaviors beyond those related to social media and will increase the social applicability of the model in the future. In the future, we will focus on the supporting evidence from social media addiction-related research rather than the empirical research on substance addiction and Internet addiction. In addition, we will provide extensive discussion and a strict definition of sample selectivity. This model is mainly based on platforms driven by visual content and public interaction (such as Instagram and TikTok), and its positive reinforcement mechanisms (such as likes and algorithm recommendations) may have a more significant effect. For platforms that mainly rely on private social interaction (such as WeChat), negative reinforcement mechanisms (such as escaping the pressure of reality) may hold greater weight, but positive reinforcement mechanisms (such as social recognition from acquaintances) still exist. It is necessary to analyze the specific usage scenarios and develop detailed models for different platform types and verify their cross-platform applicability. At the same time, the current model relies on theoretical derivation and mainly relies on indirect evidence (with limited empirical research and direct evidence related to social media addiction), which needs to be validated through future longitudinal studies. Therefore, readers and researchers who refer to this study should be cautious when discussing the resulting data.

## 9. Conclusions

This study systematically elucidated the neurobehavioral mechanism and dynamic evolution of social media addiction by constructing a dual-pathway model of positive and negative emotional reinforcement. In the early stages, dopamine-driven positive reinforcement (the pursuit of social rewards) dominates, while in long-term dependence, there is a shift to negative reinforcement (emotional avoidance) maintenance due to the degeneration of the prefrontal self-control function. During the dynamic transition from the early to late stages of addiction, positive and negative reinforcement are independent of each other and have a certain interactive effect. Individual differences, cultural values, and algorithmic exploitation further regulate the pathway strength. The integrated intervention framework proposed based on this breaks through the limitations of a single-intervention mode through the targeting of different stages and cross-system interaction and collaboration. This model not only provides an integrated explanation for the mechanism of social media addiction but also points out directions for clinical intervention and interdisciplinary policy design at different stages of addiction.

## Data Availability

No new data were created or analyzed in this study.
